# Evaluating Determinants of Environmental Risk Perception for Risk Management in Contaminated Sites

**DOI:** 10.3390/ijerph110606291

**Published:** 2014-06-16

**Authors:** Piyapong Janmaimool, Tsunemi Watanabe

**Affiliations:** 1 Graduate School of Engineering, Kochi University of Technology, Tosayamada-cho, Kami City, Kōchi 782-8502, Japan; E-Mail: tum16615@hotmail.com; 2 School of Management, Kochi University of Technology, Tosayamada-cho, Kami City, Kōchi 782-8502, Japan

**Keywords:** risk judgment, environmental risk mitigation, risk communication, risk perception

## Abstract

Understanding the differences in the risk judgments of residents of industrial communities potentially provides insights into how to develop appropriate risk communication strategies. This study aimed to explore citizens’ fundamental understanding of risk-related judgments and to identify the factors contributing to perceived risks. An exploratory model was created to investigate the public’s risk judgments. In this model, the relationship between laypeople’s perceived risks and the factors related to the physical nature of risks (such as perceived probability of environmental contamination, probability of receiving impacts, and severity of catastrophic consequences) were examined by means of multiple regression analysis. Psychological factors, such as the ability to control the risks, concerns, experiences, and perceived benefits of industrial development were also included in the analysis. The Maptaphut industrial area in Rayong Province, Thailand was selected as a case study. A survey of 181 residents of communities experiencing different levels of hazardous gas contamination revealed rational risk judgments by inhabitants of high-risk and moderate-risk communities, based on their perceived probability of contamination, probability of receiving impacts, and perceived catastrophic consequences. However, risks assessed by people in low-risk communities could not be rationally explained and were influenced by their collective experiences.

## 1. Introduction

The development of industrial sectors worldwide has contributed to vast damage to the environment and human health [1,2,3,4]. The Maptaphut industrial development area, a chemical industry hub in Thailand, is one of many cases representing a failure in environmental risk management. After the industrial estate was established, all types of environments in the area, including soil, water resources, and air, have been contaminated with hazardous substances and compounds [5,6,7,8]. The most serious issue is polluted air, which has been assumed as a cause of cancer and respiratory diseases among patients in the area [9,10]. The results of air monitoring during the 2007–2013 period showed that many types of volatile organic compounds (VOCs) in ambient air, including benzene, 1,3-butadiene, chloroform, and 1,2-dichloromethane, were above the annual standard [11]. In 2003, the National Cancer Institute in Thailand revealed that the number of cancer patients in the area was significantly higher than the national average and in Bangkok City [12]. It was also found that the rate of patients with diseases caused by environmental pollution had increased rapidly in the area since 2003 [13].

Although environmental problems caused by industrial activities in the area have been enthusiastically addressed by governments and the industrial sector, many parties are still concerned and believe that the risks associated with industrial activities still exist. One of the critical issues is a failure in risk communication among laypeople, governments, and the industrial sector. This failure has impacted the decision-making process which, until now, cannot be carried out if there is no agreement among all parties involved. Governments mostly make decisions regarding the development of industrial activities based on experts’ scientifically estimated risks; however, local residents’ risk judgments are not well understood or considered. As a result, industries have been growing despite public protests. Thus, the differences in risk judgments among laypeople, governments, and the industrial sector are a major cause of the problems in risk communication [13,14,15].

The causes determining laypeople’s risk judgments and perceptions need to be thoroughly studied in order to create effective risk communication between governments and the public [14,16,17,18]. Comprehending laypeople’s fundamental understanding of risk-related judgment can help risk communicators achieve the following: effectively establish communication efforts, properly select pieces of information and their formats [8], and foster information sharing among relevant parties. Risk perception is filtered differently by people according to their attitudes and moral values [16]. In addition to social and cultural factors (such as gender, value systems, and social norms), people’s conscious, analytical way of thinking may cause significant differences in risk perception [16]. Crawford-Brown [19] noted that residents’ perceived risks might depend on the evidence they possess regarding the frequency, severity, and variability of effects. Laypeople’s risk judgments also involve judgments of probability [15,20], severity of catastrophic consequences [20], and perceived control [20].

Currently, a range of previous, relevant research mostly explained risk perception based on the assumption that laypeople had limited scientific knowledge and capability to cope with the risks they faced; thus, their perceptions were significantly influenced by a wide spectrum of social and psychological factors such as fear, familiarity with the risk, ability to control the risk, *etc.* [21,22,23]. For example, Americans’ perceptions of the dangers of nuclear waste storage were significantly affected by psychological factors such as fear, distrust, and uncertainty [21]. However, in the present times, with the enhanced quality of education received by laypeople, an increase in public environmental awareness, the strength of laypeople’s social networks with other organizations, and varieties of public media, people’s easier access to risk-related information possibly increases their capabilities to assess the risks they face. Psychological factors might therefore be less influential. On the contrary, laypeople’s risk perceptions might be processed based on their analytical way of thinking. Factors related to the nature of risks such as perceived probability of occurrence and severity of facing risks [22,23,24] might be more powerful in predicting laypeople’s perceived risks.

This study aimed to investigate the determinants of risk perceptions held by inhabitants of industrial communities who were experiencing different levels of hazardous gas contamination, as well as to offer suggestions that could improve the current risk communication and management. The Maptaphut Industrial Estate (MIE) in Rayong Province, Thailand was selected as a case study due to the seriousness of its contamination and the need for improving risk communication and management. In determining a sampling group, the VOC and sulfur dioxide/nitrogen dioxide (SO_2_/NO_2_) contamination in the area was first reviewed to understand the degree of potential risks existing in communities. Ten industrial communities were selected and classified into the following three types in terms of the degree of contamination experienced: high-risk, moderate-risk, and low-risk communities.

In this paper, the analysis is divided into two parts. First, risk perceptions exhibited by laypeople in the three types of communities are analyzed, and their differences in risk perception are tested. Then the determinants of these people’s risk perceptions and how these differ among them are identified and investigated. Finally, the development of risk communication and management is discussed.

## 2. Theoretical Context

### 2.1. Concepts Related to Risks

Currently, risk-related concepts are diverse. According to Lash and Wynne [18], risks can be conceptualized as the probabilities of catastrophic harm caused by technological or other modernization processes. Otway and Thomas [25] mentioned at least two major risk concepts. The first is the realist approach that views risk as a physical reality that is estimated based on scientific knowledge. The second is risk as a social construct that emphasizes the contrasting definitions of risks in social reality. In other words, risk can be conceptualized into three approaches: objective, subjective, and perceptive [19]. The objective approach refers to risk as a product of scientific research conducted based on experiments and scientific methods. In contrast, the subjective approach claims that risk is not solely objective; it varies depending on people’s state of mind influenced by collective experiences, social norms, and uncertainties. In the perceptive approach, risk is defined as the set of all destructive consequences that are believed to be possible by a person who has evidence about the frequency, severity, and variability of the effects [19]. However, Fischoff [26] stated that no definition of risk is ultimately correct, since no suitable one applies to all problems. Recently, traditional risk assessment based on science alone has increasingly come into question [17] because the risks to society are exhibiting far more diverse aspects beyond the scope of scientifically estimated risks. Ropeik [17] argued that although scientific risk assessment is thoroughly conducted by using reliable methods, results will conflict with the inherent way human beings perceive risk, because how normal people live is not well understood by experts and policymakers. Many scholars are becoming increasingly interested in risk perception. Understanding how it is perceived can potentially contribute to the improvement of risk communication [14,15,27]. Furthermore, such understanding can also help mitigate underlying impacts [28,29] and support stakeholders’ long-term engagement in risk management [30].

### 2.2. Risk Perception and Risk Judgment

Risk perception is a judgment of the adverse consequences of a particular hazard and can be made by an individual, a group of people, or society [31]. The term “risk perception” generally refers to natural hazards and threats to the environment or health [16]. Risk perception can be formed based on both belief and self-appraisal [16,31,32]. Until now, four approaches have been used to study how risks are perceived. The first approach is the sociocultural paradigm, including the cultural theory of risk or simply cultural theory. Based on the cultural theory, risk perception is constructed from beliefs influenced by social forces in society [33,34]. Although it is constructed from beliefs, this sort of risk perception reflects the interests and values of each group, the diverse meanings of the term “risk” and natural phenomena within each group [31,35].

The second approach is the psychometric paradigm, which includes the psychometric model and the basic risk perception model (BRPM). The psychometric model proposed by Fischhoff in 1978 addressed how human risk perception is significantly influenced by the physical properties of risks (voluntariness, familiarity, and catastrophic consequences), as well as psychological and cognitive factors (dread, experience, benefits associated with the risks, controllability, and knowledge) [15,26]. Psychometric studies found that each type of hazard has a specific pattern of qualities related to risk perception. Some scholars working with this approach have critiqued the cultural theory. For instance, Sjoberg’s study [36] revealed the low relationship between culture adherence and risk perceptions. He explained that risk perception is related to real risks rather than cultural aspects. In 1993, Sjoberg developed his own model, the BRPM, which explains more diverse dimensions of risk perception. It adapts the psychometric dimension [37] and includes the four factors of attitude, risk sensitivity, specific fear, and trust.

The third approach is the interdisciplinary paradigm that applies several concepts to explain risk perception. Its most distinct concept is Kasperson’s social amplification of risk framework (SARF) [38], a systematic conceptualization of how scientific risk is influenced by psychological, social, institutional, and cultural processes [39]. This model explains two processes associated with risk perception: first, risk perception is affected by a variety of social processes such as social institutions’ roles in communicating risk-related information, a range of communication channels existing in societies, institutional behaviors, and sociopolitical processes; second, risk messages are interpreted and perceived by individuals or society as a whole [40].

The last approach is the axiomatic measurement paradigm that focuses on how average people subjectively transform objective risk information [41]. It is believed that risk perception is influenced by possible catastrophic consequences (fatal outcomes, mortality rates, *etc.*) and likelihood of occurrence.

Risk perception is a dynamic process that takes place in society. The factors determining risk perception can be related to all four approaches and may differ in each specific threat. In the case of environmental health risk associated with industrial development, risk perception may not only be determined by social adherence and/or emotional factors. It is also important to understand the influence of laypeople’s comprehension of the nature of risks, including probability and consequence. People need information related to the physical nature of the risk presented to them in a way they can understand.

### 2.3. Factors Determining Risk Perception

As mentioned above, risk perception can be formed based on both belief and self-appraisal. In other words, risk perception can be processed based on a rational system [22,24] or an experimental system, which includes emotion, value, and affect in risk judgments [42], and a different set of determinate factors affects perception processed through a different system. Regarding the perceived risk held by the experimental processing system, the psychometric framework has been widely used to explain the influence of psychological and cognitive factors on the risk perception of individuals with limited understanding of risk impacts [43,44,45]. The psychological and cognitive factors include controllability, experiences, perceived benefits, and concerns. Laypeople’s ability to control the risk could play a profound role in shaping risk perception. First, risks would be highly perceived if individuals feel that they have no ability to control them, for instance, risks associated with nuclear power plants or with flying in an airplane [20,26,46]. Second, previous experiences also constitute a crucial factor that might have a positive relationship with individuals’ perceived risks [47,48,49]. As stated by Paolo *et al.* [47], people smelling unfamiliar odors may exhibit a high-risk perception due to their concerns about respiratory diseases such as asthma and lung cancer. In the case of perception about the dangers of natural hazards, according to Wachinger *et al.*’s observations [50], experiences may have both positive and negative relationships with risk perceptions. With experiences of natural calamities, laypeople mostly exhibit high perception of potential disaster damages, but in some cases, risks are perceived low if people did not receive much negative impact from previous events, and the natural catastrophe does not occur often. People think that after its last occurrence, a natural disaster is unlikely to happen again in the near future. Third, perceived benefits from industrial development comprise one of the psychological factors that has been widely investigated, whether it is associated with perceived risks. Gregory and Mendelsohn [51] stated that individual risk assessment is included with the person’s perceived benefits. When technologies are perceived as highly beneficial, their risks are relatively devalued [52]. It is therefore possible that laypeople who perceive high benefits might exhibit lower perception of the risks they face. The fourth factor constitutes family concerns, which could contribute to perceived high risks. Laypeople who live in large households and/or have families with a number of children might have high concerns regarding potential impacts of contaminated air; thus, their risk perception can be perceived as high [53].

Besides psychological and cognitive factors, laypeople’s perceived risks could be constructed based on their analytical way of thinking about the nature of risks [22,24], including the perceived probability of environmental contamination, probability of receiving impacts, and perceived severity of catastrophic consequences [16,20,22,24]. The relationships between the factors related to the nature of risks and risk perception are explained in the axiomatic approach; namely, an individual’s perceived risk is influenced by the probability of its occurrence and the likelihood of a negative outcome [41]. Currently, the contribution of factors related to the nature of risks and to environmental risk perception is still unclear and scarcely investigated in previous studies. One related research conducted by Yong *et al.* [54] found that the likelihood of injury is not a significant factor contributing to perceptions of risks associated with consumer products, but the most influential factor is severity of injury. In the case of environmental health risks, Slovic [32] found that laypeople’s risk judgments are highly related to characteristics of catastrophic potential rather than probability; if there is substantial adverse damage associated with the disaster, the perceived risk is high, though there is low probability. Furthermore, many previous studies showed that laypeople’s perception of environmental risks is a function of their psychological and cognitive characteristics, but factors related to the nature of risks have less power in explaining risk perception [55,56]. However, regarding the current situation, particularly in democratic societies (where laypeople can easily access risk information due to their strong social networks with other organizations and the enhanced quality of education), the determinants of risk perception held by laypeople could be changed.

### 2.4. Study Framework and Hypotheses

**Figure 1:**
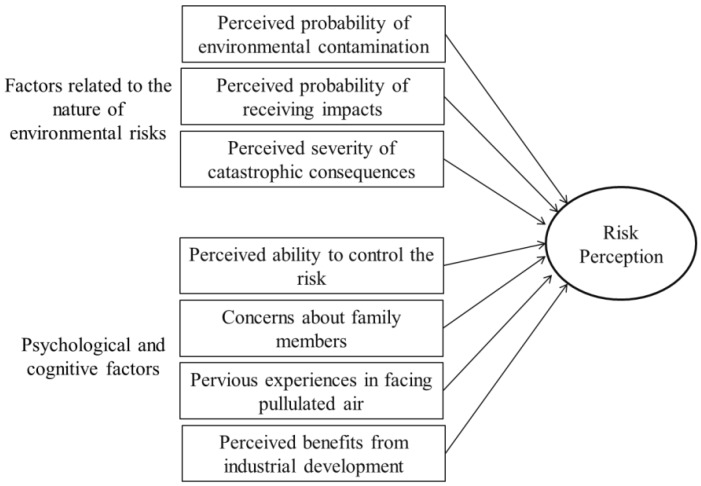
Conceptual model of risk perception.

According to the literature review, the factors potentially affecting risk perception could be divided into two main groups. The first group comprises factors related to the nature of risks, such as perceived probability of environmental contamination, probability of receiving impacts, and perceived severity of catastrophic consequences. The second group consists of psychological and cognitive factors, including perceived ability to control risks, concerns about family members, previous experiences with air pollution, and perceived benefits from industrial development. This study investigated the relationships between these selected factors and the risk perceptions held by laypeople facing different degrees of air contamination. The study defined laypeople’s risk perceptions as expected losses or potential adverse consequences caused by environmental contamination [31]. To measure risk perception, the study explored laypeople’s perceptions of the potential impacts of industrial activities on human health and well-being, which were classified into five aspects: (1) psychological effects, *i.e.*, the negative impacts of air pollutants on the human psychological system, such as anxiety or mental disorder; (2) physical health effects, *i.e.*, the impact of air pollutants on the human immunity system; (3) respiratory effects, *i.e.*, any respiratory diseases caused by inhalation of air pollutants; (4) lifestyle disruptions, *i.e.*, negative changes in local people’s daily lives, local customs, or traditions; and (5) nuisance, *i.e.*, annoying conditions caused by the changes in living environments, for example, noise pollution. Figure 1 shows an overview of the conceptual model of risk perception. Based on the assumption that laypeople are knowledgeable and have more potential to assess risk, and risk might be judged and perceived based on their rational process system rather than the experimental process system, the research hypotheses could be proposed as follows:

(1) Risk perceptions held by laypeople in three types of communities are significantly different according to the degree of air contamination experienced by each community type.
(2) Laypeople’s perceived risks are determined by factors related to the physical nature of the risks and/or psychological and cognitive factors.

## 3. Methodology

### 3.1. Case Study

The MIE in Rayong Province is one of the 29 industrial estates in Thailand. It is located at approximately 12.5° N (latitude) and 101.5° E (longitude), near the Gulf of Thailand. The project was first established in 1989 by the state enterprise, the Industrial Estate Authority of Thailand (IEAT), and the Ministry of Industry [57]. The MIE initially comprised a total area of 6.72 km^2^ that consisted of agricultural farms, wasteland, and small rural farming and fishing communities. In 2002, the area was expanded to 11.2 km^2^, and it was later found that many factories are situated in nearby residential areas [58]. Currently, there are five industrial estates in the Maptaphut area: Maptaphut, East Hemaraj, Asia, Padaeng, and RIL. About 1800 factories and a seaport are situated in the area [57]. Most of the industrial plants are petrochemical factories, coal-fired power plants, chemical fertilizer factories, and oil refineries. The industrial development in the area has been critiqued by the public due to the adverse health impacts suffered by the local people, as well as other negative social impacts, including drug abuse, crime, and pregnancy among young women [59].

Environmental problems in Maptaphut, such as polluted air, wastewater, polluted groundwater, and soil contamination, have concerned the public, industrial investors, governments, and nongovernmental organizations (NGOs). Among these problems, air contamination is perceived as the most serious one [7]. According to the results of air quality monitoring conducted by Department of Pollution Control during the 2007–2013 period, several types of VOCs were found to be above the national standard. Other air pollutants are also distributed throughout the area, including NO_2_, SO_2_, carbon monoxide (CO), and particulate matter (PM10) [7,8].

The Maptaphut municipality and neighboring areas were selected as a case study because of the seriousness of their environmental contamination and the need for risk mitigation and communication strategies. Until 2013, there were 38 communities in the Maptaphut area. The population consisted of 56,591 people (28,504 male and 28,087 female), with 42,295 households [60]. The area’s five industrial estates are surrounded by residential and commercial areas.

### 3.2. Determining the Sampling Group

A sampling group was determined based on the degree of hazardous gas contamination throughout the Maptaphut area. To classify the levels of potential threat faced by the communities, the study employed the results of Thepanondh *et al.* study [7] on VOC (benzene and 1,3-butadiene) contamination, as well as the results of Chusai *et al.* study [8] on SO_2_ and NO_2_ concentrations. The hazardous gases and compounds investigated in those two studies have been assumed to be a cause of cancer and respiratory diseases in the area [10].

Regarding the study conducted by Thepanondh and colleagues, VOC concentrations across the Maptaphut area were measured by means of gas chromatography/mass spectrophotometer (GC/MS) and conducted based on the United States Environmental Protection Agency’s toxic organic compounds (USEPA TO-15) procedure. The results showed that the VOC concentrations in the area varied according to the proximity to emission sources and types of compounds. Although this investigation was conducted during the 2007–2008 period, the results remain consistent with those of air monitoring conducted on a monthly and annual basis by Department of Pollution Control [61]. More specifically, benzene and 1,3-butadiene have thus far been found to be higher than the annual national standard. In the case of SO_2_, and NO_2_ concentrations, the study carried out by Chusai and colleagues included observations of these two compounds’ dispersion throughout the Maptaphut area by using a spatial model called the American Meteorological Society-Environmental Protection Agency Regulatory Model (AERMOD). The results showed varying degrees of NO_2_ and SO_2_ concentrations caused by both stack and nonstack sources; the differences in the findings also depended on the geographic and atmospheric conditions in each particular area.

To determine the degrees of hazardous gas and compound contaminations experienced by different areas in the Maptaphut municipality, the study employed geographic information systems (GIS) to assess contamination situations based on data provided by those two studies. The degree of concentration in each area was divided into three levels, according to the Air Quality Index (AQI) established by the USEPA [62] (see Table 1). Low concentration means that it potentially generates health impacts, and it is suggested that people with respiratory diseases, children, and the elderly avoid any outdoor activities. Moderate concentration means that it potentially generates high health impacts, and it is recommended that people with respiratory diseases avoid any outdoor activities. For general people, especially children and the elderly, outdoor exercise should be limited when high levels of pollutants are present in the air. High concentration means that it could generate severe health impacts, and it is strongly recommended for the general public to remain inside a building or shelter.

**Table 1:** Determining degrees of pollutant concentration experienced by local communities.

Type of Gas and Compound	Degree of Concentration (μg/m^3^)			National Standard *
	High	Moderate	Low	
NO_2_	500–3000	200–500	<200	320 (1 h)
SO_2_	1000–2700	600–1000	<600	300 (24 h)
Benzene	3.5–4.7	2.5–3.5	1.7–2.5	1.7 (year)
1,3 Butadiene	0.48–0.58	0.38–0.48	0.33–0.38	0.33 (year)

* According to Department of Pollution Control, Thailand.

**Figure 2:**
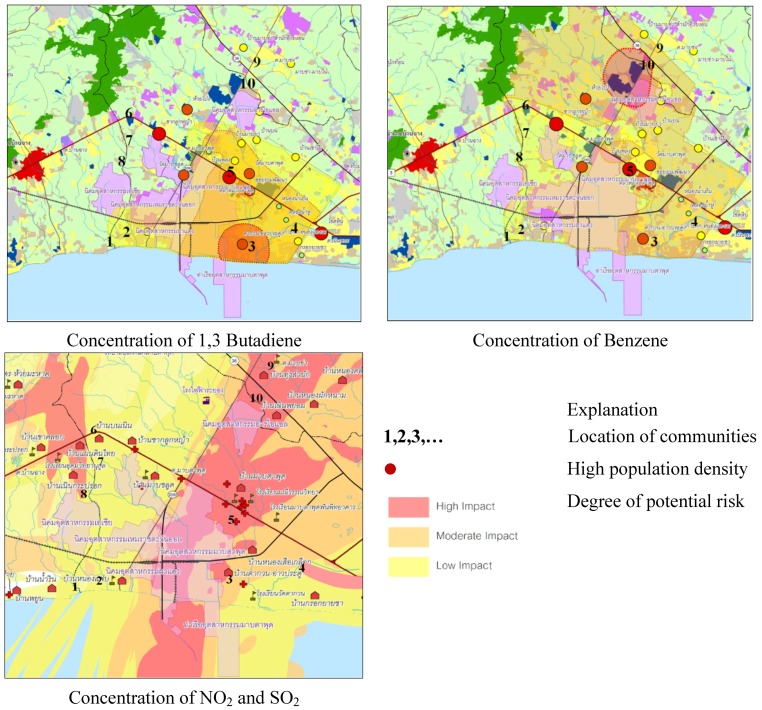
Distribution of hazardous gases and compounds throughout the Maptaphut area.

The results of the GIS analysis demonstrated the distribution of hazardous gases and compounds throughout the Maptaphut area (see Figure 2). The numbers shown in Figure 2 represent the respective locations of selected communities. Ten local communities, all of which were relatively old and established before the industrial projects, were selected for this study. These selected communities were categorized into four types, according to their respective levels of hazardous gas contamination. In classifying a type of community, communities located in areas with a high concentration of each type of hazardous gases or compounds (benzene, 1,3-butadiene, SO_2_, or NO_2)_ were given a score of 3. Communities, located in areas with a moderate concentration were assigned a score of 2, and communities located in areas with a low concentration were assigned a score of 1. A score of 0 was given to communities located in areas associated with a degree of pollutant concentration lower than the national standard. Then, the average score assigned to each community was calculated, and classified as one of the four categories such as lowest-risk community, low-risk community, moderate-risk community, and high-risk community. The results are shown in Table 2.

**Table 2:** Degrees of potential risks faced by Maptaphut communities.

Community	1,3 Butadiene Concentration	Benzene Concentration	NO_2_ and SO_2_ Concentration	Average *	Potential Risk	*N*.
1. Banprayoon and Namrin	1	1	1	1.00	low	19
2. Nuangfab	1	1	1	1.00	low	11
3. Bantrakual	3	2	2	2.33	high	20
4. Nuenpra	2	2	3	2.33	high	31
5. Maptaphut	2	1	3	2.00	moderate	40
6. Banbonnuen	0	1	2	1.00	low	14
7. Banpandintai	0	1	1	0.67	low **	8
8. Nuenkrapork	0	1	3	1.33	low	8
9. Mapkha	0	2	3	1.67	moderate	18
10. Nuenpayom	0	3	3	2.00	moderate	12
Total						181

Notes: * (0–0.75 = lowest-risk community, 0.76–1.50 = low-risk community, 1.51–2.25 = moderate-risk community, 2.26–3 = high-risk community). ** Only one community was defined as a lowest-risk community. To effectively perform the statistical analysis, the study, therefore, included this community in low-risk communities. In addition, the community is also located nearby the other low-risk communities. The degree of potential risk faced by this community might not enormously differ from those low-risk communities.

### 3.3. Data Collection and Analysis

In-depth interviews with the local people were conducted in March 2013. Then the questionnaire was created and distributed to 200 people living in the selected communities during October and November 2013. In total, 181 questionnaire sheets (about 90%) were completed. The factors, variables, and types of questions used to collect the data are shown in Table 3. The measurement of variables is presented below.

(1) Risk perception: A Likert scale, a single-select, rating scale question method [63], was used to collect the data related to respondents’ attitudes about industrial risks. Respondents were asked to rate their level of concern about potential impacts of air pollutants on their health and well-being, divided into five aspects (see Table 3). In contrast to previous research in risk perception, where the relevant characteristics of risk and rating scales have been based on literature reviews [45], this study created judgment scales reflecting degrees of risk perception based on information received from the in-depth interviews with laypeople. Based on the results of in-depth interviews, laypeople often simply exhibited degrees of concerns about potential impacts of air contaminations, such as “no impact”, “low impact”, or “high impact”. In this study, the 5- point rating scale ranged from 0 (“not at all concerned”) to 4 (“strongly concerned”). Respondents were asked nine questions, and the results obtained would be tested for their correlation before being added and calculated as a mean score, representing a level of risk perception.
(2) Factors related to the nature of risks, including perceived probability of environmental contamination, probability of receiving impacts, and severity of catastrophic consequence: These factors were measured using single-select rating questions. Based on the results of in-depth interviews with laypeople, 4-point Likert scale questions were created. Respondents were asked to rate each question, ranging from 1 (“no possibility/no severity”) to 4 (“high probability/high severity”).
(3) Psychological and cognitive factors, including perceived ability to control the risks, family concerns, previous experiences with air pollution, and perceived benefits from industrial development: To measure respondents’ perceived ability to control the risks, they were asked to rate their degree of capability in protecting themselves from the dangers of polluted air. Based on the results of in-depth interviews with laypeople, a 3-point Likert scale question was created. The rating scale ranged from 1 (“not at all”) to 3 (“highly capable”). In the case of measuring their concerns about family members, the survey simply asked about the household size. Regarding their previous experiences with air pollution, respondents were asked to indicate the frequency of feeling irritated in their eyes or nose when staying near the plants. The rating scale of frequency ranged from 1 (“never”) to 3 (“always”). To measure the factor related to perceived benefits from industrial development, respondents were asked whether their household incomes increased since the development of industrial activities in the area, and the rating scale ranged from 0 (“not at all”) to 4 (“significantly increased”).

**Table 3:** Factors, variables, and development of questionnaire.

Factors	Variables	Questions
Risk perception	Lifestyle disruption	- Have industrial activities in the area impacted your original career? - As a result of industrial development, how much can you use local resources for your leisure activities?
	Respiratory effect	- Has air quality in the area caused respiratory diseases among residents?
	Physical health effect	- Has air quality in the area caused several kinds of cancer among residents? - Has air quality in the area caused diseases related to self-immunity systems such as immunity disorder, fever, *etc.*?
	Psychological effect	- As a result of industrial development, do you feel worried about your health? - As a result of industrial development, do you feel worried about your future life in Maptaphut?
	Nuisance effect	- Have industrial activities caused nuisance such as noise or smells? - Has the current condition of the community caused nuisance such as traffic jam, congestion, noise, smells, *etc.*?
Nature of environmental risks	Probability of contamination	- What is the possibility that industries still generate polluted air in the area?
	Probability of receiving impacts	- What is the possibility that you will be impacted by air pollution in the area?
	Severity of consequences	- How severely can contaminated air in the area affect humans?
Psychological and cognitive factors	Perceived ability to control the risks	- Do you know how to protect yourselves from contaminated air?
	Concerns (number of family members)	- How many family members do you have?
	Previous experiences with air pollution	- Have you ever felt irritated in your eyes or nose when staying near the vicinity of factories?
	Perceived benefits from industrial development	- Has industrial development in the area generated more income for your family?

All the collected data were statistically analyzed by using two methods. First, the analysis of variance (ANOVA) was performed to identify the significant differences in risk perception of people living in high-risk, moderate-risk, and low-risk communities. Next, to identify the factors determining the risk perception of people living in each type of community, a multiple regression analysis was performed in order to evaluate the relationship between risk perception (dependent variable) and selected potential predictive factors (independent variables), such as the physical nature of risks, perceived ability to control the risks, family concerns, and previous experiences. The results are presented as a set of regression equations describing the statistical relationship between the dependent and independent variables. Finally, all results are discussed in terms of their implications for the development of risk communication strategies.

## 4. Findings and Discussion

### 4.1. General Characteristics of Respondents

The number of respondents comprised 51 from high-risk communities, 70 from moderate-risk communities, and 60 from low-risk communities. Overall, the number of male respondents was slightly higher than that of female respondents, at 51.4% and 48.6%, respectively. Table 4 shows the general characteristics of respondents in the three types of communities. The distributions of gender, age, and educational levels were not significantly different, based on the results of the Chi-square test. Most of them were within the working age range; respondents in the 30–39 and 20–29 age groups occupied a major proportion of those living in the moderate-risk and low-risk communities. Most of the respondents in the high-risk communities belonged to the 30–39 and 40–54 age brackets. Regarding their educational levels, the majority of the respondents in the three communities only finished high school, which is considered sufficiently eligible for several kinds of low-skilled jobs such as those in the service and industrial manufacturing sectors, construction work, as well as labor in the agricultural sector. The survey also showed that the careers and incomes of the respondents in the three types of communities were significantly different, according to the results of the Chi-square and ANOVA tests. Most of the respondents in the high-risk communities worked as industrial employees and in private companies, respectively. The majority of the respondents in the moderate-risk and low-risk communities were laborers in the agriculture and service sectors, with relatively lower incomes than their counterparts in the high-risk communities.

**Table 4:** General characteristics of respondents.

Characteristic		High-risk Community [*N* = 51]		Moderate-risk Community [*N* = 70]		Low-risk Community [*N* = 60]		Test Statistics
		*N*/Mean	%	*N*/Mean	%	*N*/Mean	%	
Gender	Male	30	58.8	36	51.4	27	45.0	*X*^2^ = 2.109
	Female	21	41.2	34	48.6	33	55.0	
Age	Under 20 years old	3	5.9	8	11.4	7	11.7	*X*^2^ = 9.613
	20–29	12	23.5	27	38.6	13	21.7	
	30–39	17	33.3	18	25.7	20	33.3	
	40–54	15	29.4	11	15.7	12	20.0	
	55 and above	4	7.8	6	8.6	8	13.3	
Education	Primary school	5	9.8	8	11.4	8	13.3	*X*^2^ = 4.982
	High school	28	54.9	41	58.6	31	51.7	
	Vocational degree and Associate degree	3	5.9	3	4.3	5.0	8.3	
	Undergraduate degree	13	25.5	18	25.7	13	21.7	
	Higher than undergraduate degree	2	3.9	0	0.0	3	5.0	
Career	Public servant	8	15.7	4	5.7	6	10.0	*X*^2^ = 19.956 *
	Laborer in agriculture sector and service sector	6	11.8	28	40.0	23	38.3	
	Industrial worker	13	25.5	10	14.3	8	13.3	
	Private company employee	10	19.6	5	7.1	6	10.0	
	Self-employed, such as business owner, service provider, and merchant	8	15.7	16	22.9	10	16.7	
	Other	6	11.8	7	10.0	7	11.7	
Income	Average income/month (Thai Baht ± SD)	14,458 ± 6774.86		11,464 ± 4547.91		11,650 ± 7546.6		*F* = 3.908 *

Note: * *p* < 0.05.

### 4.2. Laypeople’s Risk Perceptions

Table 5 shows the mean scores of the risk perception variables and their correlations. Respondents exhibited higher perceptions of physical health effect, nuisance, and respiratory effect than those of psychological health impacts and lifestyle disruption. The results of the Pearson correlation analysis revealed that most of the perception variables were positively correlated with one another. The results of Bartlett’s test of sphericity and the Kaiser-Meyer-Olkin (KMO) measure of sampling adequacy also manifested high correlations among all variables, indicating that all these variables can represent a degree of risk perception. All variables were added and calculated as a mean score representing a degree of risk perception. Higher scores represented higher perceived risks. The Table 6 shows an average risk perception score and descriptive statistics of potential predictors. Generally, it was found that all factors related to the nature of risks are more correlated with laypeople’s risk perception than psychological and cognitive factors.

The mean scores of perception of environmental risks exhibited by respondents from high-risk, moderate-risk, and low-risk communities were compared, and the differences among the groups were statistically proven by the results of the one-way ANOVA. First, the test of homogeneity of variances showed unequal variances across groups (*sig* = 0.001). Therefore, the results of Welch’s t-test were used instead of the regular ANOVA test. The findings showed that the degrees of risk perception significantly differed among respondents living in different communities facing varying levels of hazardous gas contamination, *F* (2178) = 12.908, *p* = 0.000, η_p_^2^ = 0.138. Because of the unequal variances across groups, a post-hoc analysis using Dunnett T3 was then performed to demonstrate multiple comparisons (see Table 7).

**Table 5:** Mean scores of risk perception variables and their correlations.

Variable		Lifestyle Disruption		Psychological Impacts		Respiratory Impact	Physical Health Impact		Nuisance	
		1	2	3	4	5	6	7	8	9
1	Have industrial activities in the area impacted your original career?	1.000								
2	As a result of industrial development, how much can you use local resources for your leisure activities?	0.439 **	1.000							
3	As a result of industrial development, do you feel worried about your health?	0.309 **	0.529 **	1.000						
4	As a result of industrial development, do you feel worried about your future life in Maptaphut?	0.427 **	0.464 **	0.614 **	1.000					
5	Has air quality in the area caused respiratory diseases among residents?	0.170 *	0.353 **	0.645 **	0.504 **	1.000				
6	Has air quality in the area caused several kinds of cancer among residents?	0.204 **	0.372 **	0.552 **	0.522 **	0.701 **	1.000			
7	Has air quality in the area caused diseases related to self-immunity systems such as immunity disorder, fever, *etc.*?	0.124	0.381 **	0.523 **	0.506 **	0.689 **	0.773 **	1.000		
8	Have industrial activities caused nuisance such as noise or smells?	0.234 **	0.442 **	0.469 **	0.458 **	0.511 **	0.515 **	0.595 **	1.000	
9	Has the current condition of the community caused nuisance such as traffic jams, congestion, noise, smells, *etc.*?	0.226 **	0.291 **	0.252 **	0.247 **	0.276 **	0.275 **	0.327 **	0.644 **	1.000
Mean		2.24	2.36	2.57	2.40	2.71	2.77	2.82	2.85	2.61
SD		1.152	1.059	0.924	0.993	0.868	0.920	0.885	0.853	0.934

Notes: * *p* < 0.05, ** *p* < 0.01. Bartlett’s test of sphericity = 806.773 d*f* = 36 *p =* 0.000. Kaiser-Meyer-Olkin (KMO) measure of sampling adequacy = 0.847.

**Table 6:** Average risk-perception score and descriptive statistics of potential predictors.

Items		Mean/*N* (%)	SD	Correlation with RP
Risk perception (RP)	Risk perception (RP)	2.604	0.665	1
Factors related to the nature of environmental risks	Perceived probability of environmental contamination	3.381	0.661	0.415
	Perceived probability of receiving impacts	3.293	0.705	0.404
	Perceived severity of catastrophic consequences	3.265	0.712	0.339
Psychological and cognitive factors	Perceived ability to control the risk			0.043
	- NeverNot at all	39(21.5%)	--	
	- NeverModerately capable	117(64.6)	--	
	- NeverHighly capable	25(13.9)	--	
	Concerns about family members	4.133	1.912	−0.205
	Pervious experiences with air pollution			0.222
	- Never	29(16%)	--	
	- NeverSometimes	109(60.2%)	--	
	- NeverOften	43(23.8%)	--	
	Perceived benefit from industrial development	2.276	1.221	0.243

**Table 7:** Differences in means of risk perception scores given by respondents in three types of communities.

Type of Community	*N*	Mean	SD	Mean Difference (Multiple Comparison)		
				High-risk Communities	Moderate-risk Communities	Low-risk Communities
High-risk	51	2.96	0.759	--	0.38989 *	0.62775 *
Moderate-risk	70	2.57	0.601	−0.38989 *	--	0.23786 *
Low-risk	60	2.34	0.501	−0.62775 *	−0.23786 *	--
Total	181	2.60	0.665			

Notes: (Welch’s t-test analysis) *F* = 12.908, *p* = 0.000 * The mean difference is significant at 0.05.

The results indicated that the average risk perception score given by respondents in low-risk communities was significantly lower than those in moderate-risk (*p* = 0.045) and high-risk communities (*p* = 0.000). Similarly, respondents in moderate-risk communities had significantly lower risk perception scores than those in high-risk communities (*p* = 0.009), but higher than those in low-risk communities. The risk perception scores given by the respondents showed that those in high-risk and moderate-risk communities believed that the existence of industrial risks was still high and would potentially bring significant losses to their lives. In contrast, respondents in low-risk communities exhibited low risk perception, which signified minimal expected losses caused by air contamination in the area.

An interpretation of the analysis results could be that risks perceived by laypeople are related to the degrees of hazardous gas contaminations estimated by experts [7,8]. The results of this analysis could support Sjoberg’s [36] claim that the relationship between cultural adherence and risk perception was low, and laypeople’s perceptions were significantly related to real risks. In this study, which emphasized environmental health risks, the cultural theory [34,37] might not be an appropriate concept to explain how environmental health risks are determined by laypeople. Although most of the respondents in this study shared a similar culture, they had significantly different degrees of risk perception.

### 4.3. Factors Determining Risk Perception

Multiple regression analysis was performed to test if the factors related to the nature of environmental risks and psychological factors significantly predicted respondents’ risk perceptions. The predictors were the seven indices, while the criterion variable was the degree of risk perception. The results indicated that the linear combination of the seven predictors could predict the degree of risk perception exhibited by respondents, but its power to explain the degrees of risk perception held by the respondents in the three types of communities was different (see Table 8). In high-risk communities, the linear combination of the selected predictors was significantly related to the degree of risk perception, *F*(7,42) = 9.655, *p* = 0.000. The multiple correlation coefficient was 0.785, indicating that approximately 61.7% of the variance in risk perception can be accounted for by the linear combination of selected predictors. The linear combination of these predictors could also explain a significant proportion of the variance in the risk perception score given by respondents in moderate-risk communities (*R*^2^ = 0.456, *F*(7,62) = 7.415, *p* = 0.000) and low-risk communities (*R*^2^ = 0.414, *F*(7,52) = 5.258, *p* = 0.000).

**Table 8:** Summary of regression analysis for variables predicting environmental risk perception.

Variable	High-risk Community [*N* = 50] Missing 1				Moderate-risk Community [*N* = 70]				Low-risk Community [*N* = 60]			
	*B*	SE B	β	VIF	*B*	SE B	β	VIF	*B*	SE B	β	VIF
Perceived probability of environmental contamination	0.534	0.185	0.395 *	2.057	0.119	0.095	0.128	1.184	0.091	0.093	0.126	1.470
Perceived probability of receiving impacts	0.165	0.168	0.139	2.181	0.359	0.083	0.451 *	1.246	0.042	0.090	0.060	1.458
Perceived severity of catastrophic consequences	0.178	0.162	0.133	1.589	0.223	0.083	0.271 *	1.150	0.001	0.086	0.001	1.305
Perceived ability to control the risks	−0.184	0.144	−0.132	1.163	−0.002	0.098	−0.002	1.034	0.005	0.096	0.005	1.046
Concerns about family members	−0.021	0.034	−0.063	1.124	−0.034	0.039	−0.090	1.173	−0.033	0.028	−0.128	1.041
Previous experiences with air pollution	0.026	0.126	0.021	1.112	−0.022	0.105	−0.020	1.014	0.408	0.085	0.522 *	1.052
Perceived benefits from industrial development	0.207	0.054	0.398 *	1.174	0.068	0.050	0.130	1.051	0.101	0.057	0.195	1.063
*R*^2^	0.617				0.456				0.414			
*F*	9.655 *				7.415 *				5.258 *			

Note: * *p* < 0.01.

The significance of individual variables in predicting risk perception scores is presented in Table 8. It was found that the variables significantly predicting risk perceptions held by the respondents in the three types of communities were different. For respondents in high-risk communities, two of the seven predictors were statistically significant: perceived probability of environmental contamination and perceived benefits from industrial development. In contrast, the perception score given by respondents in moderate-risk communities was significantly predicted by the variables of perceived probability of receiving impacts and perceived severity of catastrophic consequences. The perception score given by respondents in low-risk communities was significantly predicted by only one predictor: perceived experiences with air pollution in the area. Table 8 presents a regression model with significant predictors of risk perception held by respondents in each type of community.

Based on the findings, environmental risks were determined differently by respondents who lived in the three different types of communities. Similar to what Aven [31] addressed, this study found that respondents may either use beliefs or self-appraisal to judge and perceive risks. The risk perceptions of respondents from high-risk and moderate-risk communities have been proven as significantly related to how they think about the nature of risks. This finding is partly related to the results of Slovic’s [20] and Leiserowitz’s research [24], which suggested the influence of the nature of risks on the public’s environmental risk perceptions. Respondents in high-risk communities judged risks based on their perceived probability of environmental contamination; however, respondents in moderate-risk communities assessed risks by considering the probability of being impacted by the contamination, as well as the potential adverse impacts they might face. On the other hand, the perceptions exhibited by respondents from low-risk communities were not particularly determined by factors related to the nature of risks, but were instead significantly influenced by one of the psychological and cognitive variables, that is, previous experiences with air pollution. Possibly, the perceptions of residents in low-risk communities were not processed based on the rational system but formed based on their beliefs, which were affected by previous experiences.

Besides being determined by perceived probability of contamination, the risk perceptions of respondents in high-risk communities were also significantly influenced by their perceived benefits generated from industrial development in the area. This finding is related to those of the studies conducted by Slovic [64] and Gregory and Mendelsohn [51], which also stated the influence of perceived benefits on perceived risks; however, the positive relation between perceived benefits and perceived risks found in this study was unexpected and different from the results of previous studies [51,52]. For instance, Gregory and Mendelsohn [51] concluded that individual risk assessment is included with one’s perceived benefits, whereas Alhakami and Slovic [52] argued that when technologies are perceived as highly beneficial, risks are relatively devalued. In this study, respondents in high-risk communities seemed to understand that the more benefits they gained, the more risks they faced, whereas respondents in the other two types of communities did not include benefits at all in their risk assessments and perceptions. This situation could be explained that most of respondents in high-risk community work in the industrial complex (see Table 4), and relatively have higher income than those respondents from moderate-risk and low-risk communities. It is possible that respondents in high-risk communities are certain that there are potential risks associated with industrial activities, and they tend to accept those risks as long as benefits are gained.

Overall, the results indicated that laypeople used different processing systems to judge and perceive risks. Moreover, the factors related to the physical nature of environmental risks played more important roles in shaping the risk perceptions of laypeople in high-risk and moderate-risk communities than psychological and cognitive factors did. Possibly, people became more knowledgeable, and thus they judged risks based on their rational processing system [22,24].

## 5. Implications for Development of Risk Communication

Generally, the study implies that laypeople living in contaminated sites are knowledgeable, since the respondents’ degrees of risk perception are related to the levels of hazardous gas and compound concentrations estimated by experts. Additionally, laypeople are not emotional when judging and perceiving risks. As evidenced by the findings, most of the psychological factors are not associated with perceptions of environmental risks. Risk is determined based on laypeople’s understanding of the nature of environmental risks, such as perceived probability of contamination caused by industrial activities, perceived probability of receiving impacts, and perceived severity of catastrophic consequences. With the exception of residents in low-risk communities, the respondents’ perceived risks are formed based on their experiences with air pollution. Inhabitants of low-risk communities may possibly pay less attention to facing risks that are less serious for them. However, this particular case may not be applicable in explaining the risk perceptions of people in every contaminated site, since this study’s participants have been struggling with environmental problems for a long time and have exerted much effort in fighting against organizations that have failed to manage risks. Furthermore, they have been educated with a variety of information and have gained many experiences.

Additionally, the study demonstrates that perceived benefits generated by industrial activities are not considered when risks are judged by respondents in moderate-risk and low-risk communities. As for study participants in high-risk communities with commercial areas, they have realized the correlation between gaining substantial benefits and taking high risks. In this regard, the institutions involved may be unsuccessful in their efforts to mitigate the public’s perceived risks by merely providing different types of compensation and facilities without demonstrating an initiative to effectively minimize risks. Reducing or increasing people’s risk perceptions significantly depends on how they understand the nature of risks. Communicating information related to the physical nature of risks is therefore vital; on the contrary, poor communication can lead to high public anxiety and high risk perception.

This study also helps relevant parties identify the gaps in risk perception when laypeople’s fundamental understanding of risk-related judgment is compared to those of other stakeholders. If the causes of the risk perception gap among parties are accurately indicated, then risk communication strategies, including the goals and methods of communication efforts, as well as information types and formats, can be properly designed to bridge this gap [65,66]. This study’s results suggest that appropriate information, such as knowledge of community sensitivities that influence the public’s perceived probability of receiving impacts, should be mutually exchanged among involved parties. Laypeople with a solid understanding of such sensitivities can play a crucial role as messengers. Two-way or collaborative communication between and among stakeholders should therefore be established. Moreover, due to the diverse risk perspectives among residents of different types of communities, those in high-risk and moderate-risk communities might be more interested in information about the nature of environmental risks, such as the probability that industries might cause contamination, the amount of pollutants released, and the potential of contracting diseases. Scientific data regarding the nature of risks can gain higher acceptance among people in high-risk and moderate-risk communities but might be completely rejected by inhabitants of low-risk communities. Therefore, in designing an effective environmental risk communication, the broad range of the public’s risk judgments should be seriously taken into consideration.

## 6. Conclusions

The study presents how environmental risks are determined by lay people living in contaminated sites, and how risk communication can be created based on lay cognitive models. First, the study found that the degree of risk judged by lay people was related to the degree of hazardous gas contamination existing in their living area. This implies that lay people’s risk perception can reflect what risk actually is in reality. Factors related to the physical nature of risks play an important role in shaping the risk perception of people living in areas with high and moderate concentrations of hazardous gas; however, people living in an area with low concentrations of hazardous gas judge and perceive risk based on their experiences. In addition, perceived benefit from industrial development in the area was not taken into account when risk was determined by people in moderate-risk and low-risk communities, while people in high-risk communities appeared to understand that the more benefits they received, the more risk they faced. This finding implies that the effort to provide affected communities with some sort of compensation as well as facility without demonstrating an effort to mitigate risks might not be able to reduce laypeople’s perceived risk. Additionally, to effectively communicate risks with lay people and to support an effective environmental risk management, the study suggests that laypeople’s risk information interpretations should be clearly understood by relevant parties, so that what laypeople are concerned can be taken into account in risk management. In addition to merely providing laypeople with risk information, an environmental risk communication should emphasize fostering information sharing between laypeople and relevant parties and putting an effort to include laypeople in risk management process. The study also suggests that people with different risk perspectives need to be communicated with using different information formats. People who judge risks based on belief may completely deny scientific information related to the nature of risk, while such information might be accepted by people who perceive risks based on their self-appraisal.
